# Similar Quality of Visual Working Memory Representations between Negative and Positive Attentional Templates

**DOI:** 10.5334/joc.380

**Published:** 2024-07-16

**Authors:** Matthieu Chidharom, Mahsa Zafarmand, Nancy B. Carlisle

**Affiliations:** 1 Department of Psychology, Lehigh University, Bethlehem, Pennsylvania, 18015, USA

**Keywords:** Distractors, color-wheel, attentional templates, inter-individual differences, fluctuations

## Abstract

Visual working memory (VWM) plays an important role during visual search, with some theories suggesting an equivalence between working memory representations and guidance from attentional templates. However, recent work has shown that participants can also use ‘negative templates’, the foreknowledge of distractor-features stored in VWM, to guide attention away from distractors during visual search. These negative templates must also be represented in working memory, but the question remains whether the quality of the working memory representations underlying negative and positive templates are similar, in spite of their opposite impacts on attention. In this study, participants (N = 33) engaged in a visual search task for a shape-defined target after receiving a positive cue (target color), negative cue (distractor color) or neutral cue (non-informative). In 20% of the trials, a color-wheel probe was presented instead of a search array to measure the quality of the cue representation stored in VWM. Our results revealed that participants were more likely to guess in response to neutral cues than negative cues. Yet, the comparison between positive and negative cues showed no significant differences. However, we found no difference in memory precision for the three cue types. More interestingly, the more the VWM quality is boosted by the negative cue, the greater the ability to guide attention away from distractors. Such a pattern of results might map to recent evidence of between-individuals differences in utilization of negative cues. These findings highlight the distinction between attentional templates and simple maintenance in working memory.

## 1. Introduction

Many theories of attention suggest that the control of attention is dependent upon visual working memory (Desimone and Duncan, 1995; Bundesen et al., 2005; Wolfe et al., 1989). For example, when we are looking for tomato sauce at the grocery store, we are able to use our knowledge of features of our target item (e.g., red) to generate internal positive templates that guide our search toward likely target items. The Biased Competition Theory (Desimone & Duncan, 1995) makes the most direct linkage between working memory and attention by suggesting that holding a representation in working memory is sufficient to guide attention to matching items in the visual scene. According to this proposal, holding an item in working memory will lead to continued activity in cells which are tuned toward the working memory representation. When multiple stimuli are presented in a visual scene, objects that match working memory receive a combination of activation driven from the working memory maintenance as well as the incoming sensory activation. This increased activation creates a bias to attend memory-matching items, and is proposed to be the neural instantiation of a positive target template. So, according to Biased Competition, attentional templates are the working memory representations, and working memory maintenance is inextricably linked to attentional template generated attentional biases (Carlisle, 2019). This viewpoint has received much support in the literature (Soto, et al., 2008; Olivers, et al., 2011), and it is still widely accepted that items in working memory will automatically bias visual attention (Oberauer, 2019).

However, recent evidence has also shown that foreknowledge of distractor items, also called negative templates, can allow us to guide attention away from distractor items to improve visual search efficiency. The first study explicitly directed at assessing negative templates was conducted by Arita and colleagues (2012; but see Carlisle & Woodman, 2011; Sawaki & Luck, 2010, and Woodman & Luck, 2007). In Arita’s study, participants searched for a shape-defined target in a display containing two colors of Landolt Cs. Each search display was preceded by a cue that could indicate the upcoming distractor color (negative cues), indicate the upcoming target color (positive cues) or be non-informative (neutral cues). Note that the colors cued changed on each trial, meaning that the colors would need to be maintained in working memory to serve as templates. In this design, both negative and positive cues lead to faster RTs compared to neutral cues, but with smaller benefits for negative compared to positive cues. These benefits from negative templates have now been replicated multiple times (Carlisle & Nitka, 2019; Conci et al., 2019; Reeder et al., 2017, 2018; Zhang et al., 2020; 2022) and are derived from *ignoring* items that match working memory.

To reconcile these results with proposals of biased competition (Desimone & Duncan, 1995) and viewpoints suggesting an automatic link between working memory and attention (Soto et al., 2008; Olivers et al., 2011), some recent frameworks hypothesize that negative templates will automatically guide attention towards distractors, then are rapidly suppressed in a reactive manner, as proposed by the “search and destroy” hypothesis (Moher & Egeth, 2012). However, other perspectives, like the “active suppression” hypothesis (Arita et al., 2012) consider these findings on negative templates difficult to fully reconcile with biased competition theory, and instead suggest negative templates can proactively guide attention away from distractor features in a preventative manner (Geng & DiQuattro, 2009; Chidharom & Carlisle, 2023).

If negative templates in working memory lead to avoiding attention to memory-matching items, while positive templates in working memory lead to attention toward these items, one important outstanding question is how the working memory representations are similar or different for positive and negative templates? This question is important for our understanding of the mechanisms underlying attentional templates, but also in evaluating the theoretical proposals suggesting automatic links between working memory and attention.

Previous work has utilized neurophysiological measures of working memory to examine the relationship between attentional templates and working memory. One neurophysiological index of the active maintenance of object representations in VWM is the contralateral delay activity (CDA), an event-related potential observed on lateral occipital-temporal electrodes (Vogel and Machizawa, 2004; Vogel et al., 2005). Carlisle and colleagues (2011) first used the CDA to demonstrate that attentional templates are maintained in working memory when the attentional template changes on each trial, providing physiological evidence in humans that working memory is employed to maintain positive templates prior to visual search. Following a similar logic, Rajsic and Woodman (2020) used the CDA to contrast the working memory maintenance of positive and negative templates. They found both positive and negative cues lead to similar amplitude CDA responses, suggesting that both positive and negative attentional templates are stored in VWM. However, these results do not tell us about the quality of the working memory representations of positive compared to negative templates.

**Figure 1:**
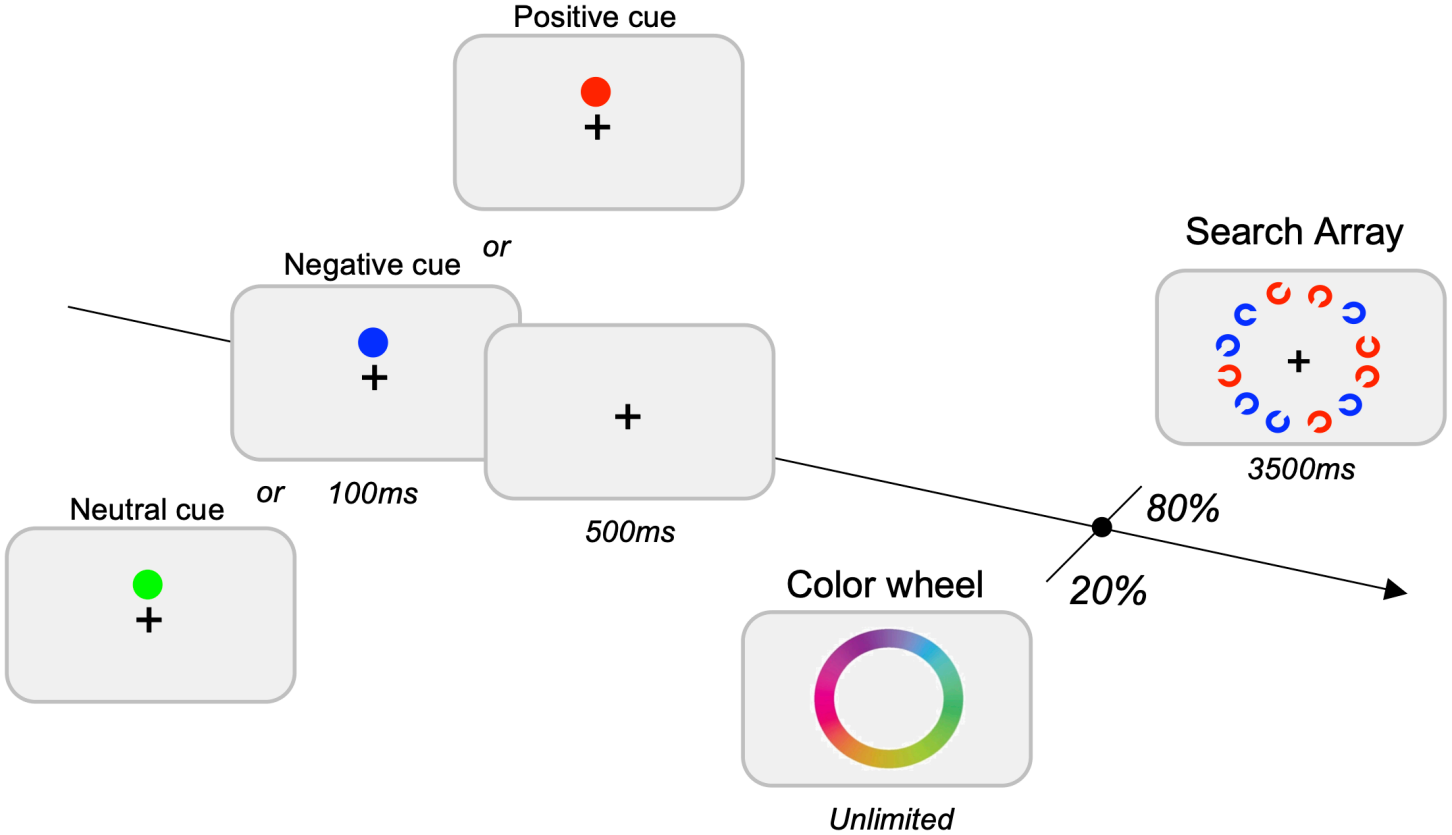
**Design of the Visual Search Task**. In separate blocks, neutral, negative, or positive cues were presented with randomized color selection per trial. Search trials constituted 80% of the task, requiring participants to locate gap-oriented up or down Landolt-C targets. Additionally, 20% of trials were probe trials, prompting reporting of the preceding cue’s color on a color-wheel.

In order to gain a more precise measure of the quality of the working memory representation, one can use the color wheel method of testing working memory, as introduced by Zhang and Luck in 2008. The color wheel task serves as a valuable tool for obtaining a detailed assessment of WM capabilities. Within this task, participants are instructed to select the color on the color wheel that closely corresponds to the given cue (see Figure 1). When participants have forgotten the item in memory, they will report a random color on the wheel, leading to a uniform pattern of guesses. When the participant maintains the item in memory, the reported color should be close to the correct position on the wheel, although responses will likely fall in a normal distribution centered on the correct color. By measuring the standard deviation of this distribution of responses, we can determine the precision of the memory representation. Small standard deviations indicate very precise working memory representations, while larger standard deviations indicate less precise working memory representations.

Previous studies have used this method to examin the relationship between working memory quality and the efficiency of visual search for positive templates. Rajsic and colleagues (2017) had subjects report the color of a positive cue on a color wheel following a search array. Positive templates were recalled with greater precision and were less likely to be forgotten compared to a baseline condition, where the color in working memory was not to be used as a visual search template. Even more interestingly, Rajsic and colleagues separated trials with “good” and “bad” memory quality by using a median split on VWM performance. They revealed that search was faster during “good” compared to “bad” VWM states, suggesting a critical role of VWM precision in visual search efficiency. Although a similar pattern of results has been observed recently by Dube and Al-Aidroos (2019) for positive templates, there are at present no studies examining the working memory representations underlying negative (or distractor) templates.

One possibility is that both positive and negative templates must be maintained equally well in working memory, in order to use this information to guide search. This would be in line with recent evidence suggesting that working memory representations are distinct from attentional templates (Yu, et al., 2022, Kerzel, 2019, Olivers and Eimer, 2011). Another possibility is that the working memory representations of negative templates must be degraded, either by reducing the likelihood that they are maintained or by reducing the precision of the memory, in order to limit their impact on attention. This would be more compatible with Biased Competition (Desimone & Duncan, 1995) and associated viewpoints (Soto, et al., 2008; Olivers, et al., 2011), but still difficult to reconcile with the concept that what is maintained in working memory leads to an automatic attentional bias towards items matching working memory.

Hollingworth (2022) highlights the inherent conflict between the concept of negative templates (Carlisle, 2023) and the prevailing viewpoint that working memory leads to automatic attentional biases towards memory matching items. It is therefore critical to understand more about the working memory representations underlying negative templates and contrast them with the representations of positive templates. The goal of this study is therefore to directly contrast the working memory precision and likelihood of maintenance of positive and negative templates. To do so, we engaged participants in a visual search task similar to that used by Arita and colleagues (2012). In the majority of trials, participants engaged in a visual search task. We randomly intermixed a memory probe on 20% of trials, where instead of a search array participants were presented with a color wheel and asked to report their memory of the color cue. If representations in working memory are automatically linked to attentional biases, we would expect negative templates to show a lower precision or decreased likelihood of maintenance compared to positive templates. However, if working memory representations are fundamentally separated from attentional templates (Yu, et al., 2022; Carlisle, 2019), we would expect to see a similar precision and likelihood of maintenance for positive and negative templates. Finally, if a high-quality VWM representation is critical to generate an effective attentional template, as suggested by the findings in positive cues from Rajsic and colleagues (2017), we would expect to observe higher negative cue benefits when the VWM quality is higher at the intra- and inter-individual levels.

## 2. Methods

### Participants

We analyzed a sample of 33 undergraduates from Lehigh University who gave informed consent and participated in a search task for course credit (Mean Age = 19, SD = 0.84, 9 females). Procedures were approved by Lehigh University IRB. All participants reported normal or corrected-to-normal vision and normal color perception.

### Stimuli

Stimuli were presented on a gray background (90.0 cd/m^2^) at a viewing distance of ap- proximately 60 cm. The search cue was a filled colored circle (1.3°) presented at the center of the screen. Search items were outlined circles (1.3° in diameter with a 0.2° line thickness) with a gap (0.5° long) that were presented 6.3° from fixation. The colors appearing for the cue and during the search array were randomly selected on each trial from all colors used in the continuous report color wheel created by Suchow et al., (2013; memtoolbox). Within a trial, each color was at least 60° away from each other and no memory colors were repeated. The target-color items and the distractor-color items were spatially intermixed.

### Procedure

The trials start with the presentation of a fixation cross on a gray background. After 500 ms, a cue was presented for 300 ms and characterized by a filled circle color cue. The cue was followed by a 500 ms fixation screen. In 80% of the trials, a 12- item visual search array of Landolt-Cs was presented on an imaginary circle centered on the fixation cross. Two colors were selected for the search array at random on each trial from all colors used in the continuous report color wheel. The subjects had to detect a target characterized by a gap opening facing the top or bottom of the Landolt-C (see Figure 1). They were instructed to press the up arrow of the keyboard when the gap was at the top, and the down arrow when the gap was at the bottom. The search array remained on the screen until 200 ms after response, or for a maximum of 3,500 ms. In 20% of the trials, the color wheel was presented for participants to report the memorized color of the cue. Reports were made by selecting from a continuous report color wheel (Suchow et al., 2013; Lively et al., 2021; Zhang & Luck, 2009). The wheel was randomly rotated in each trial. Participants had no time limits to report the memorized color.

This task was divided into 3 blocks of 90 trials (18 color wheel probes/block), separated by cue type. In the positive cue block, the cue indicated the color the target would appear in the upcoming block. Using this cue would mean participants would only need to search through the 6 items in this color to find the search target. Similarly, the negative cue indicated that the cued color would *not* be the target, allowing participants the possibility of ignoring the 6 distractor items appearing in the negative cue color. Finally, the neutral cue block contained a cue that would not appear in the upcoming array, providing no information to help complete the search task. The order of the blocks were randomized across participants, and participants received instructions about the meaning of the cue and practice trials for the cue before beginning each experimental block (Figure 1). A feedback regarding memory accuracy was given during the practice trials only.

### Data and Statistical Analysis

**Figure 2:**
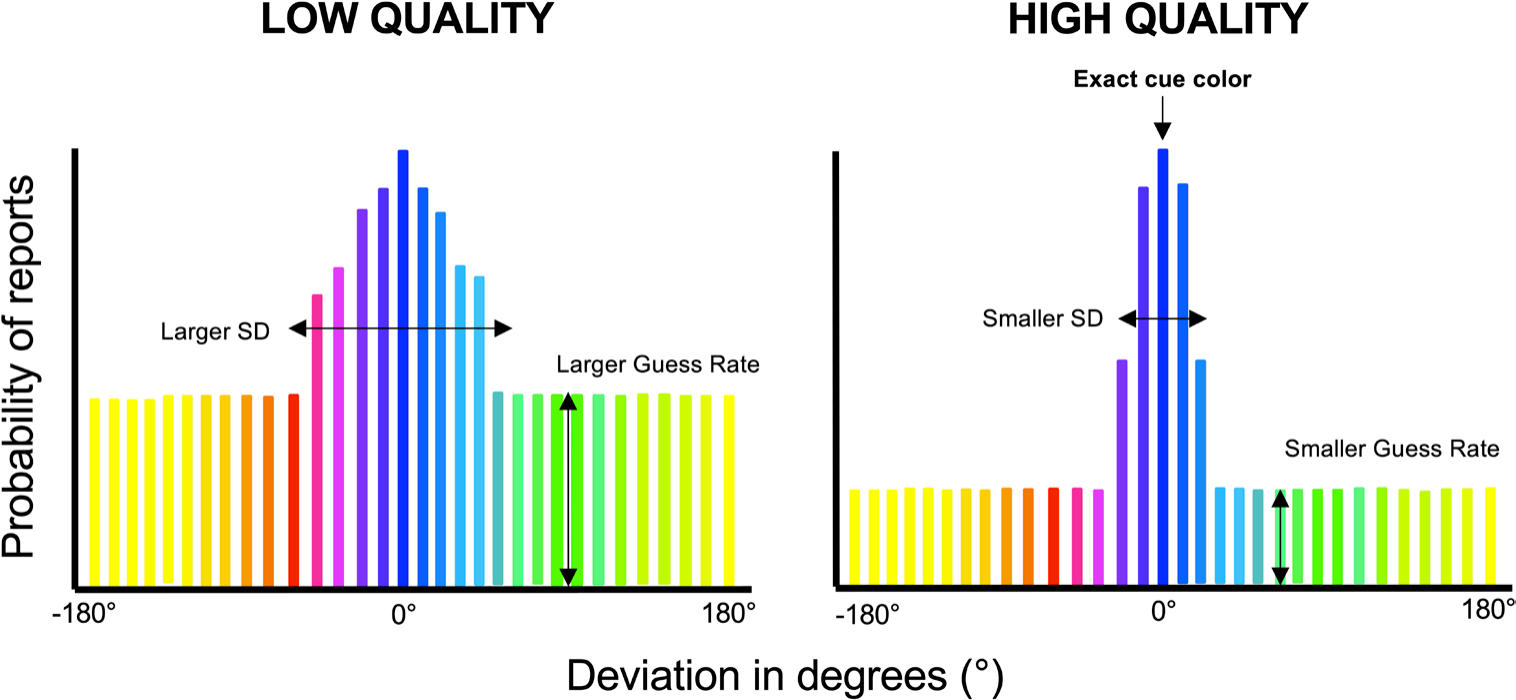
**Measures of the Visual working memory quality: An example for the negative cue (blue) condition regarding** Figure 1. If working memory is linked to an automatic attentional guidance towards memory-matching items, we would anticipate that measures of working memory quality (Zhang & Luck, 2008) would show evidence of lower quality representations for negative templates than positive templates. This would appear as a larger standard deviation and/or higher guess rate for negative templates than positive templates.

One participant was excluded for bad accuracy (lower than 2.5 standard deviations from the mean accuracy). On the search-trials, trials with RT less than 300 ms, trials with no response during the search window, or with incorrect responses were excluded from the analysis. The percentage of correct responses is the number of correct responses divided by the total number of trials per condition, multiplied by 100. On the probe-trials, visual working memory performance was accessed using response error that is the angular deviation between the selected and the original cue color. Performance was further quantified by fitting a simple mixture model to the distribution of response errors for each participant using MemToolbox (Suchow et al., 2013). We modeled the distribution of response errors as the mixture of a von Mises distribution centered on the correct value and a uniform distribution. We obtained maximum-likelihood estimates for two parameters: the dispersion of the von Mises distribution (SD), which reflects the quality of the participant’s memory; and the height of the uniform distribution (g), which reflects the probability of guessing (indicators of the VWM quality are illustrated Figure 2). ANOVAs were performed including the within-subject factors Cue (Neutral, Positive and Negative). In the case of statistically significant interactions, paired *t-tests* were conducted. The hypothesis, task design and statistical analysis plan were pre registered on Open Science Framework (https://osf.io/qse8y).

## 3. Results

### 3.1 Search trials

**Figure 3:**
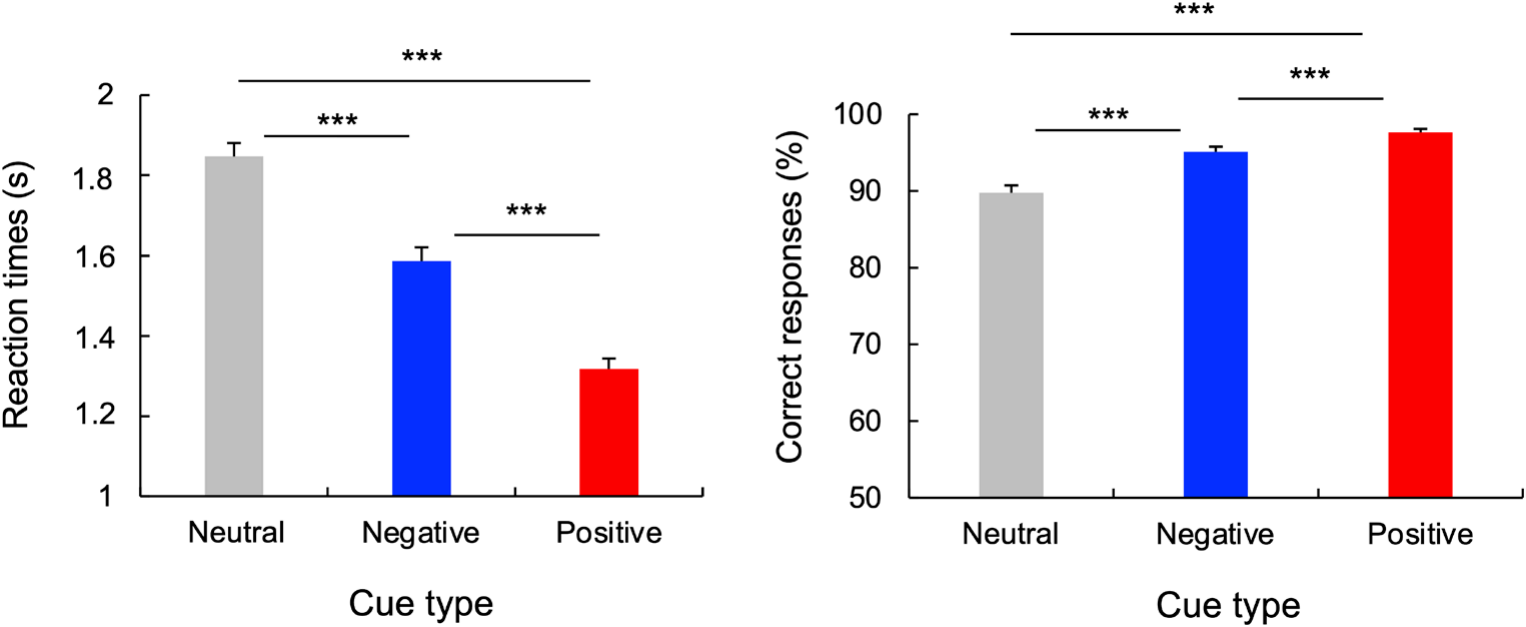
**Performance on search trials**. Error bars represent between-subject standard error from the mean RTs. ***p < 0.001.

The ANOVA performed on mean RT revealed a main effect of Cue, *F*(2,32) = 120.226, *p* < 0.001, *η_p_^2^* = 0.79. Post-hoc paired t-tests revealed faster RT for positive (1317 ms) compared to negative (1586 ms), *t*(32) = 9.24, *p* < 0.001, and neutral cues (1846 ms), *t*(32) = 15.11, *p* < 0.001. Moreover, the RT for the negative cue was faster compared to the neutral cue, *t*(32) = 6.86, *p* < 0.001 (Figure 3).

The ANOVA performed on the correct responses revealed a main effect of Cue, *F*(2,32) = 35.671, *p* < 0.001, *η_p_^2^* = 0.535. Post-hoc comparisons revealed a higher rate of correct responses to negative cues compared to neutral cues, *t*(32) = 4.81, *p* < 0.001. Moreover, participants had more correct responses to positive cues compared to negative *t*(32) = 4.33, *p* < 0.001 and neutral cues *t*(32) = 7.9, *p* < 0.001 (Figure 3).

### 3.2 Probe trials

#### Performance on the probe trials is shown in Figure 4

**Figure 4:**
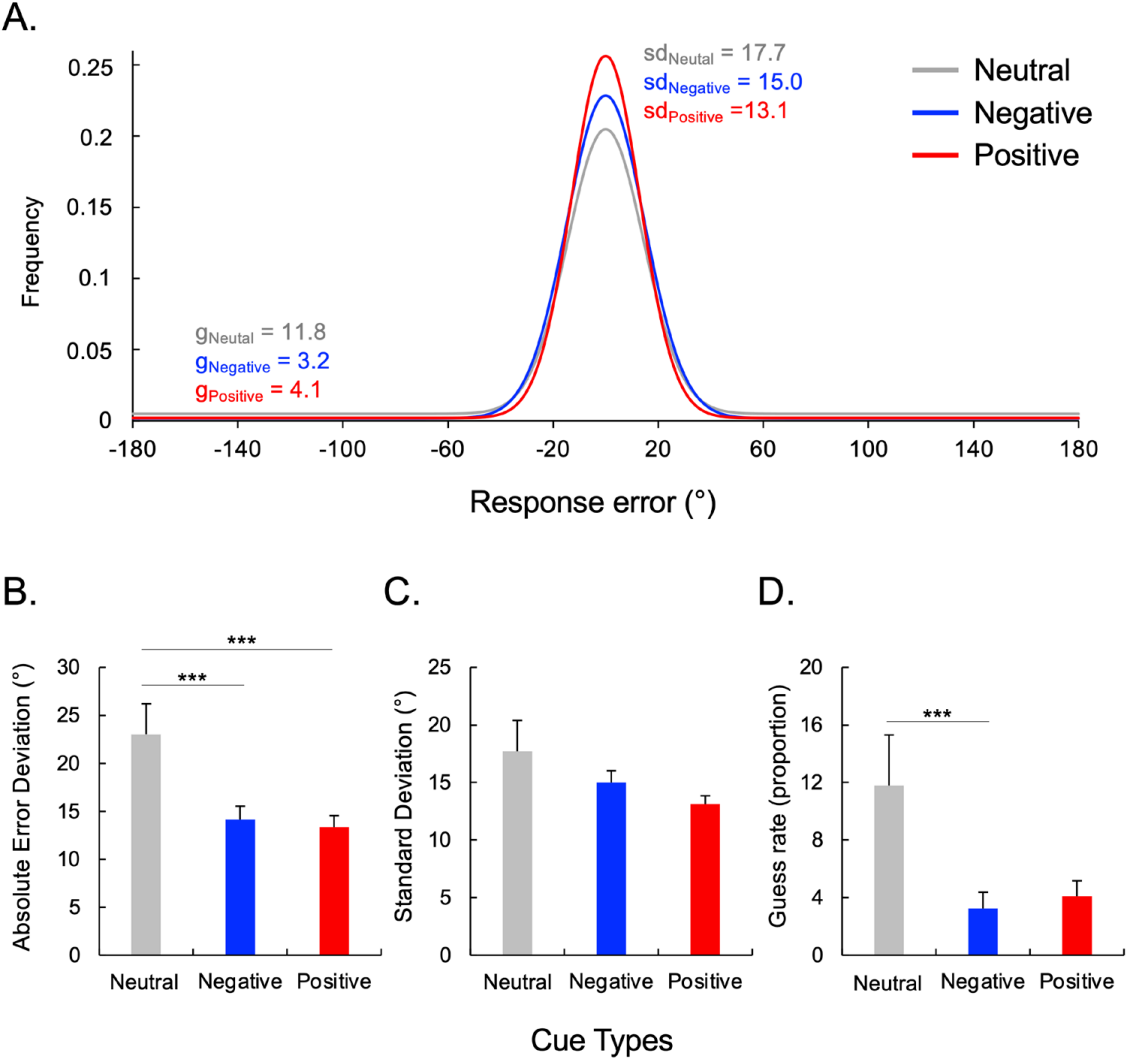
**Performance on probe trials. (A)** Distributions of recall errors with the fits of Zhang and Luck’s (2008) standard mixture model across the three cue conditions. **(B)** Absolute error deviation of reported value. **(C)** Standard Deviation estimates from the mixture model. **(D)** Guess rate estimates from the mixture model. Error bars represent between-subject standard error from the mean. ***p < 0.001.

The ANOVA revealed a main effect of Cue on mean absolute error deviation, *F*(2, 31) = 11.23, *p* < 0.001, *η_p_^2^* = 0.266. The post-hoc tests revealed that the mean absolute error deviation was lower after both negative and positive cues compared to neutral cues, *t*(31) = 3.43, *p* = 0.002, and *t*(31) = 3.53, *p* = 0.001, respectively. However, no difference was observed between negative and positive cues, *t*(31) = 0.727, *p* = 0.473. In order to better understand these differences in working memory, we next utilized the mixture model (Zhang & Luck, 2009) to separate the distribution into precision of items maintained in memory and likelihood of maintaining an item in memory (Figure 4B).

The ANOVA revealed no main effect of Cues on SD (*p* = 0.128) (Figure 4C).

The ANOVA revealed a main effect of Cue on the guess rate, *F*(2, 32) = 5.47, *p* = 0.006, *η_p_2* = .015. Participants’ guess rates were higher in neutral cues compared to negative, *t*(32) = 2.60, *p* < .001. However, no significant differences in guess rate were observed between neutral and positive cues *t*(32) = 1.2, *p* = .103, and between negative and positive cues (*p* = 0.222) (Figure 4D).

### 3.3 Exploratory analysis: Pearson’s Correlations

#### Interindividual differences in VWM in attentional template benefits

**Figure 5:**
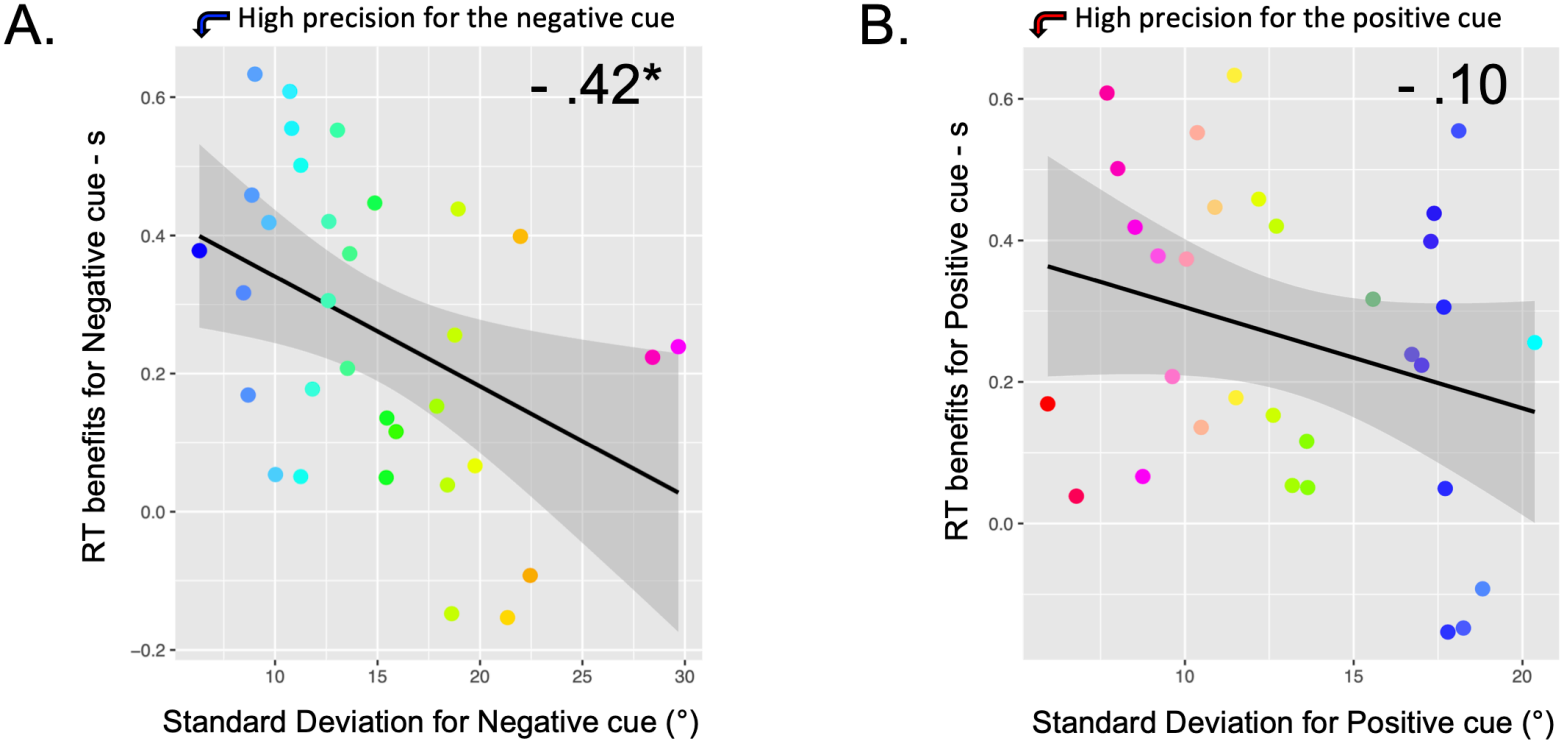
**Correlation between memory precision and benefits of negative and positive cues. (A)** Individuals with higher VWM precision (lower s.d.) showed larger negative cue benefits. **(B)** No relationship between VWM precision and positive cue benefits. *p < 0.05.

In order to examine whether there is a relationship between working memory precision and visual search efficiency, we next examined the relationship between standard deviation estimates from the mixture model and benefits from the negative and positive cues. The benefits were computed by subtracting the reaction times (RTs) for neutral cues from the RTs for informative cues (negative or positive cues). The result of the Pearson Correlation showed a significant negative relationship between the Benefits of RT for negative cues and the variability of the error deviation of negative cues, *r* = –0.42, *p* = 0.017 (Figure 5). No effect was observed between the RT benefits and the variability of the error deviation for positive cues (*r* = –.098, *p* = 0.593). This indicates individual differences in working memory precision can predict attentional efficiency.

#### State-based differences in VWM in attentional template benefits

Since it has been shown that VWM quality fluctuates over time between good and bad states (deBettencourt et al., 2019), we wanted to explore if search performance was related to the VWM states. To do so, we used the same methods as Rajsic and colleagues (2017) and performed a median split on the absolute memory error deviation. This approach allows us to explore the search performance on the search trial (N–1) preceding the color wheel probe (N) during periods of good and bad VWM. To explore the effect of VWM states on RT, we performed repeated measures ANOVA including the within-subject factors Cue (Neutral, Positive and Negative) and VWM states (Good and Bad VWM). In the case of statistically significant interactions, paired *t-tests* were conducted. Additionally, we conducted t-tests against zero to assess the significant RT benefits associated with both negative and positive cues. This approach was taken because ANOVA may not be suitable for revealing significant costs and/or benefits on performance associated with the VWM states.

As shown previously, the ANOVA performed on RT revealed the main effect of Cue, *F*(2,62) = 37.59, *p* < 0.001, *η_p_2* = 0.55. However, no main effect of VWM states (*p* = 0.568), nor Cue × VWM States interaction (*p* = 0.670) was observed.

The ANOVA performed on the RT benefits revealed a main effect of Cue, *F*(1,31) = 45.54, *p* < 0.001, *η_p_2* = 0.60, with higher benefits for positive compared to negative cues. However, the ANOVA did not reveal a main effect of VWM states (*p* = 0.553), nor Cue × VWM States interaction (*p* = 0.515).

**Figure 6:**
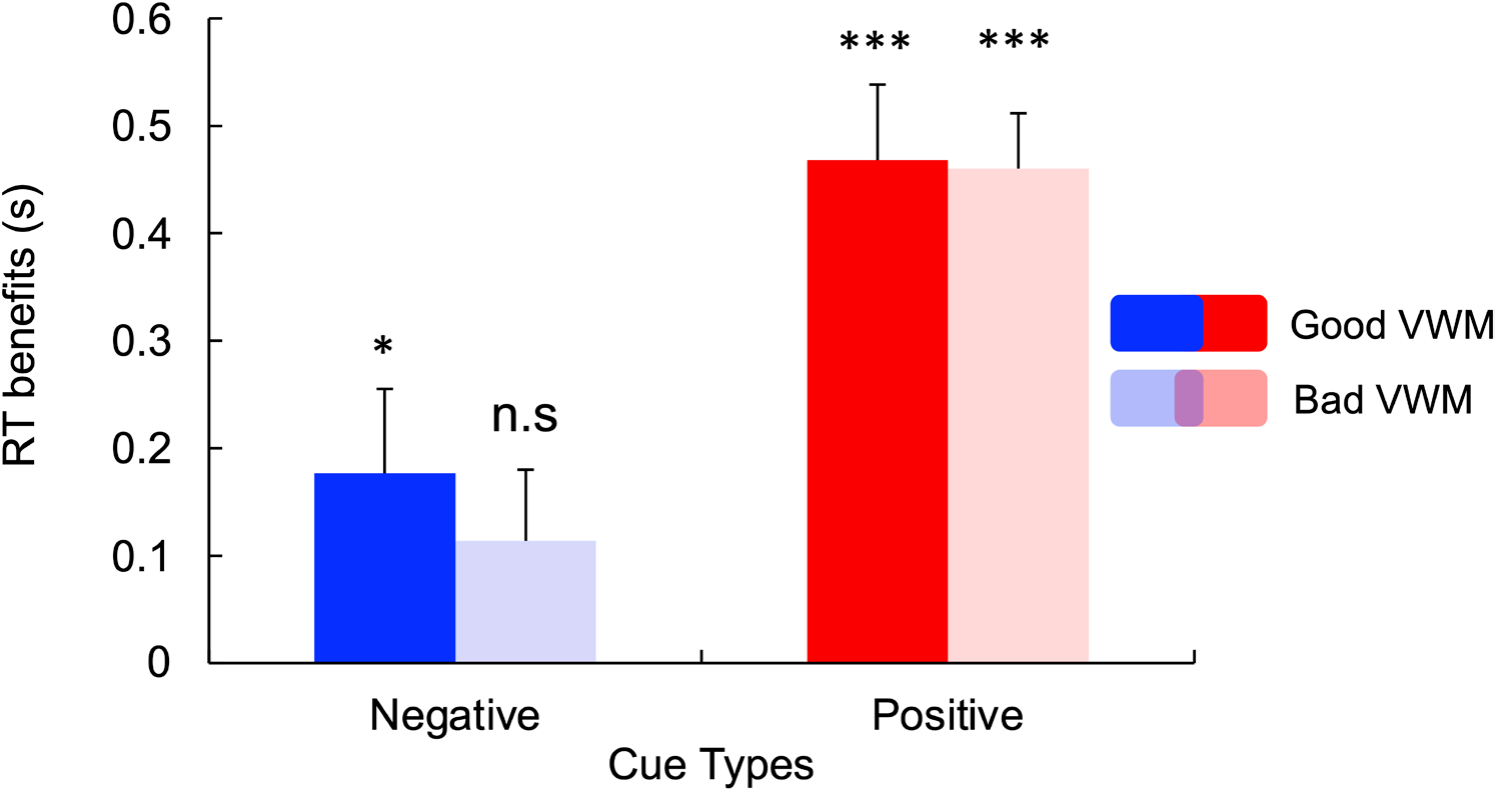
**t-tests compared to a zero baseline of the RT benefits of negative and positive cues during periods of good and bad visual working memory**. No benefits of negative cues during periods of lower VWM quality. Error bars represent between-subject standard errors from the mean RT benefits. *p < 0.05, ***p < 0.001.

To verify the benefits induced by positive and negative cues on RT, planned comparisons against zero were performed (Figure 6). After positive cues, benefits were observed during both good VWM periods, *t*(31) = –6.55, *p* < 0.001, *d* = –1.16, and bad VWM periods, *t*(31) = –8.80, *p* < 0.001, *d* = –1.56. However, after negative cues, benefits were observed during good VWM periods, *t*(31) = –2.21, *p* = 0.034, *d* = –0.39, but not during bad VWM periods (*p* = 0.101). These findings indicate that state-level differences in working memory precision influence the attentional impact of negative templates.

## 4. Discussion

Theories of attention propose a strong connection between working memory and attentional templates (Wolfe, 1989; Bundesen, 1990; Bundesen et al., 2005), with Biased Competition Theory (Desimone & Duncan, 1995) and the related “search and destroy” hypothesis (Moher & Egeth, 2012) suggesting an automatic link between maintaining an item in working memory and an attentional bias towards memory matching items (Soto, et al., 2008; Olivers, et al., 2011). Recent work on negative templates, where attention is directed away from items matching working memory, presents a serious challenge to this prevailing viewpoint (Hollingworth, 2022). The goal of this study was to better characterize the quality of the working memory representations underlying negative templates, and contrast these representations to those used to create positive templates. To do so, we used color-wheel probe trials intermixed with the typical visual search task.

Negative templates were not less likely to be maintained in working memory than the positive templates, so any differences in attentional impact cannot be explained by a reduced maintenance of negative templates vs. positive templates. We also found no significant difference between the VWM precision of negative cues, positive cues, and neutral cues, suggesting that differences in the quality of the working memory representations cannot explain the differential attentional impact of the different template types. More interestingly, the more precise the VWM for negative templates, the greater the ability to guide attention away from distractors. Such a pattern of results was observed at the state-level, by contrasting periods of high and low memory precision within the same individual, as well as at the between-subject level, by contrasting individuals with higher and lower average working memory precision. These findings reveal the critical importance of VWM quality in the use of negative templates and develop a more accurate picture of the mechanisms underlying the use of negative templates.

### Rethinking the theories and hypotheses of visual search

These results also help to refine theories on visual search and do not support the idea of an automatic link between maintenance in working memory and attentional biases, as proposed by the biased competition theory (Desimone & Duncan, 1995). Indeed, according to the Biased Competition (Desimone & Duncan, 1995) and associated viewpoints, working memory representations of negative templates might have been degraded, either by reducing the likelihood that they are maintained or by reducing the precision of the memory, in order to limit their impact on attention. However, the present study reveals a quality of the working memory representation similar between negative and positive templates, and that negative templates are actively encoded in working memory, and not degraded. This work supports an additional step in creating an attentional template beyond just maintenance in working memory (Carlisle & Woodman, 2011; Carlisle, 2019; Kerzel, 2019; Olivers and Eimer, 2011; Yu, et al., 2022). While high quality memory representations for negative cues would fit with the search and destroy model (Moher & Egeth, 2012), which suggests attention is first directed towards negatively cued items before they are suppressed, multiple studies examining the search and destroy hypothesis have failed to find evidence for this early attention towards negatively cued items in terms of fMRI (Reeder, et al., 2018), EEGs (Carlisle & Nitka, 2019), attentional probes (Zhang, et al., 2020), and eye tracking (Zhang, et al. 2022; Beck, et al., 2018). Therefore, our results are much more in line with the “active suppression” hypothesis, suggesting that the suppression of distractors occurs through a top-down engagement of attentional control actively guided by a qualitative representation of the negative template (Carlisle, 2023).

### Negative template representations are less likely to be forgotten

The analysis of memory parameter estimates revealed that participants were less likely to forget negative templates compared to neutral cues. Indeed, lower guess rates were observed after negative cues compared to positive and neutral cues. This could suggest that negative templates required greater activation and maintenance in working memory in order to be implemented. This interpretation aligns with the additional cognitive effort required to implement these negative templates (Rajsic, et al., 2020), as evidenced by the increase in fronto-medial theta oscillatory activity observed in the EEG data (de Vries et al., 2019; Chidharom & Carlisle, 2023). However, regarding the memory precision (SD), our mixture model did not reveal any boost in the precision of the visual working memory for negative or positive cues compared to neutral cues. Previous work from Rajsic and colleagues (2017) reported higher VWM precision after positive cues than their baseline task, leading us to expect a similar pattern. One possible explanation is the baseline (i.e., neutral) condition used in their task. Indeed, in the tasks used by Rajsic and colleagues (2017), the participants always had to maintain two items in working memory, only one of which served as an attentional template. In contrast, in our design the neutral (baseline) condition only a single item was presented. These differences in task demands across the two studies may tie into known differences in working memory based on the number of items maintained. According to one perspective, the flexible resource theory (Bays & Husain, 2008; Palmer, 1990; Wilken & Ma, 2004), allocating resources to several items will lower the quality of the VWM representations. Similarly, research on attentional templates suggests placing an item in a ‘prioritized’ state which can guide attention (Olivers, et al., 2011), and leads to stronger working memory representations for that item compared to items maintained in an accessory state which does not impact attention (Dube, et al., 2017; Dube & Al-Aidroos, 2019). We can thus speculate that the baseline used by Rajsic and colleagues (2017), in which two items have to be encoded, is more likely to reduce the visual working memory precision than our single-item neutral condition baseline. These increased working memory demands in Rajsic and colleagues’ work may have also led to a significant relationship between working memory and search performance, which we did not observe in our study.

### The visual working memory quality is associated with better use of negative templates

Our exploratory analysis revealed that individuals with higher VWM precision are more likely to benefit from negative templates, as revealed by the significant correlation between the memory SD and the RT benefits after negative cues. This is in line with a recent study, based on a large sample size of 54 participants, in which we revealed that individuals with higher VWM ability (measured through the k-score during a change localization task; Zhao et al. 2022) are specifically faster at using negative cues (Chidharom & Carlisle, 2024a). These results could suggest that interindividual differences in the quality of the VWM exist and play a critical role in the ability to guide attention away from cued-distractors.

While inter-individual differences in VWM seem associated with better suppression of distractors, a similar pattern of results has been observed within-individuals, at the state-level. Indeed, by separating periods of “good” and “bad” states of memory (Rajsic et al. 2019), our analysis revealed that participants benefit from negative templates during periods of “good” visual working memory but not during periods of “bad” visual working memory. This could suggest that fluctuations in VWM quality (deBettencourt et al., 2019) impair the ability to use negative templates. Interestingly, such fluctuations are not associated with positive template use since benefits of positive cues have been observed during both “good” and “bad” states of memory.

Taken together, those results based on interindividual differences and state-based differences highlight the critical role of the VWM quality to efficiently guide attention away from irrelevant items. Indeed, it has been previously shown that negative templates rely on higher proactive control mechanisms. For example, higher fronto-parietal theta power has been observed after negative cues compared to both positive and neutral cues (de Vries et al., 2019; Chidharom & Carlisle, 2023). We also recently showed that individuals with higher proactive control efficiency are better at using negative templates (Chidharom & Carlisle, 2024b).^1^ Taken together, this work suggests a coherent tripartite system to optimally suppress distractors: (1) negative templates – (2) visual working memory – and (3) proactive control. The current results are thus in line with previous research showing the critical role of working memory in efficient engagement of proactive control (Redick, 2014; Gonthier et al. 2019; Lin et al. 2022; Wiemers & Redick, 2018). To go further, we can speculate that negative templates relate to the quality of VWM representation allowing a higher efficiency in individuals’ ability to proactively avoid distractor-features and guide attention away from irrelevant items.

Most importantly, our findings draw a clear separation between working memory and attentional guidance, providing a stark contrast to a predominant view in the literature that working memory leads to an automatic attentional bias towards memory-matching items (Desimone & Duncan, 1995; Soto, et al., 2008; Olivers, 2011; Oberauer, 2019). Our study shows that similar quality working memory representations can either lead to attentional enhancement or attentional avoidance. These provide further support for the idea that attentional templates are distinct from working memory representations, an idea that has been previously suggested in the literature (Carlisle, 2019; Hollingworth, 2022; Dube, et al., 2016; Carlisle & Woodman, 2011).

## 5. Limitations

Several limitations can be drawn in the current study. First, the exploratory results on the state-based difference in visual search efficiency need to be replicated in future studies. While our ANOVA did not reveal any significant interaction between cues RT benefits and VWM states, our follow-up t-tests against zeros revealed preserved benefits for negative cues only during periods of good VWM. While we contend the against-baseline tests provide complementary information about the presence or absence of benefits within a condition, the lack of an interaction effect indicates limited differences between states when considering our experimental manipulations. A second limitation is related to the reliability of our estimates. The estimation performed by the mixed model was based on a limited number of trials (18 trials per cue conditions), and a replication with a larger number of trials is necessary to confirm these results and to enhance the robustness and reliability of findings.

## 6. Conclusions

In conclusion, our study revealed the clear role of the VWM quality in using and implementing negative attentional templates efficiently. However, there was no evidence that the working memory representations of negative templates were reduced in precision or likelihood of maintenance compared to positive templates. Future studies should better characterize the neural mechanisms underlying the quality of the VWM and how it interacts with top-down processes, especially proactive control mechanisms, to optimally avoid distractor items.

## Data Accessibility Statement

The datasets are available on Open Science Framework (https://osf.io/mr85k/?view_only=None).

## Footnotes

The correlation between SD-RT and the benefits of negative cues and SD-RT and the benefits of positive cues differed significantly (*z* = –2.60, *p* < .01).
